# Patient-specific quantification of cardiorespiratory motion for cardiac stereotactic radioablation treatment planning

**DOI:** 10.1016/j.hroo.2024.03.006

**Published:** 2024-03-21

**Authors:** Adrian Petzl, Karim Benali, Nicolas Mbolamena, Katia Dyrda, Léna Rivard, Sebastian Seidl, Raphaël Martins, Martin Martinek, Helmut Pürerfellner, Martin Aguilar

**Affiliations:** ∗ Electrophysiology Service, Department of Medicine, Montreal Heart Institute and Université de Montréal, Canada; † Department of Cardiac Electrophysiology, Saint-Etienne University Hospital, France; ‡ Department of Internal Medicine 2/Cardiology, Ordensklinikum Linz Elisabethinen, Linz, Austria; § Department of Cardiac Electrophysiology, Rennes University Hospital, France

**Keywords:** Cardiac radioablation, Ventricular tachycardia, Stereotactic body radiation therapy, Cardiorespiratory motion, 3D mapping system

## Abstract

**Background:**

Cardiac radioablation is a new treatment for patients with refractory ventricular tachycardia (VT). The target for cardiac radioablation is subject to cardiorespiratory motion (CRM), the heart’s movement with breathing and cardiac contraction. Data regarding the magnitude of target CRM are limited but are highly important for treatment planning.

**Objectives:**

The study sought to assess CRM amplitude by using ablation catheter geometrical data.

**Methods:**

Electroanatomic mapping data of patients undergoing catheter ablation for VT at 3 academic centers were exported. The spatial position of the ablation catheter as a function of time while in contact with endocardium was analyzed and used to quantify CRM.

**Results:**

Forty-four patients with ischemic and nonischemic cardiomyopathy and VT contributed 1364 ablation lesions to the analysis. Average cardiac and respiratory excursion were 1.62 ± 1.21 mm and 12.12 ± 4.10 mm, respectively. The average ratio of respiratory to cardiac motion was approximately 11:1. CRM was greatest along the craniocaudal axis (9.66 ± 4.00 mm). Regional variations with respect to respiratory and cardiac motion were observed: basal segments had smaller displacements vs midventricular and apical segments. Patient characteristics (previous cardiac surgery, height, weight, body mass index, and left ventricular ejection fraction) had a statistically significant, albeit clinically moderate, impact on CRM.

**Conclusion:**

CRM is primarily determined by respiratory displacement and is modulated by the location of the target and the patient’s biometric characteristics. The patient-specific quantification of CRM may allow to decrease treatment volume and reduce radiation exposure of surrounding organs at risk while delivering the therapeutic dose to the target.

**Key Findings**

▪ Cardiorespiratory motion of the heart is primarily determined by respiratory motion, with an average ratio of ∼11:1 compared with cardiac motion.
▪ Amplitude of cardiorespiratory motion is greatest in craniocaudal direction as opposed to anteroposterior and mediolateral, and apical segments have significantly larger motion amplitude compared with basal segments.
▪ Better understanding of the magnitude and directionality of cardiorespiratory motion may help in optimizing the patient-specific target volume delineation for cardiac radioablation.

## Introduction

Catheter ablation is a standard treatment for refractory ventricular tachycardia (VT).^1^ However, despite considerable improvements, VT recurrence rates remain significant.^1^ Radiofrequency catheter ablation can be limited by the endo/epicardial anatomy, the presence of endocardial thrombus, and/or calcifications and the inability to access deep midmyocardial circuits. Moreover, the procedural risk can be prohibitive in some patients. Recently, cardiac stereotactic body radiation therapy (SBRT) has emerged as an encouraging alternative for patients with VT refractory to or with contraindications for medical therapy or catheter ablation procedures.^2^, ^3^, ^4^, ^5^, ^6^, ^7^

However, cardiac radioablation generates several new challenges. A major consideration in any form of radiation treatment is to deliver the therapeutic energy dose to the target while sparing surrounding healthy tissues.^5^ The target for cardiac radioablation moves with breathing and cardiac contraction, resulting in complex cardiorespiratory motion (CRM) of the target. In most practices, the clinical target volume is expanded to ensure that the target VT circuit remains within the radiation field throughout the cardiorespiratory cycle.^8^ However, this empiric strategy likely unnecessarily exposes surrounding tissues to radiation. Alternatively, strategies to compensate for cardiorespiratory displacement such as gating, breath-holding, or fiducial marker tracking have been proposed to account for respiratory motion but are not yet mainstream.^9^ Importantly, the magnitude of CRM in patients with ventricular arrhythmias has not been characterized.

Understanding the determinants of CRM in this patient population may allow a reduction in treatment volume while still delivering the therapeutic dose of radiation to the target. It could also orient the development of new strategies to compensate for CRM during cardiac radioablation treatments. In this study, we aimed to quantify the magnitude and directionality of CRM of different cardiac target locations in patients with either ischemic cardiomyopathy (ICM) or nonischemic cardiomyopathy (NICM) undergoing catheter ablation for VT, who would typically be considered for SBRT.

## Methods

We retrospectively included 44 patients with ICM or NICM and recurrent VT who underwent endocardial and/or epicardial VT catheter ablation at 3 academic centers (Montreal Heart Institute, Canada; Ordensklinikum Linz Elisabethinen, Austria; Rennes University Hospital, France). The indication for catheter ablation was VT refractory to antiarrhythmic drug therapy for all cases. The ablation strategy was at the operator’s discretion as was the decision to use sedation (free breathing) vs general anesthesia (mechanical ventilation). All cases were performed utilizing CARTO 3 V7 (Biosense Webster).

### Statistical analysis

Proportions of categorical variables were reported in percentage. Mean ± SD was reported for all continuous variables. Vectorial data were transformed into absolute values, to represent motion amplitude correctly.

To test for significant differences between groups, chi-square or Fisher’s exact tests were performed for categorical variables, and Student’s *t* test or analysis of variance was performed to compare means of normally distributed continuous variables or Kruskal-Wallis test in non-normally distributed data. Tukey’s multiple comparison test was used for post hoc testing between groups after performance of analysis of variance. Multiple linear regression was used to estimate the relationship of dependent variables on independent variables (cardiac motion and respiratory motion). *P* values ≤.05 were considered statistically significant (we tested for 2-sided significance for all analyses except chi-square and Fisher’s exact tests). Statistical analyses were performed with Prism 8 (GraphPad Software).

### CRM analysis

**Figure 1:**
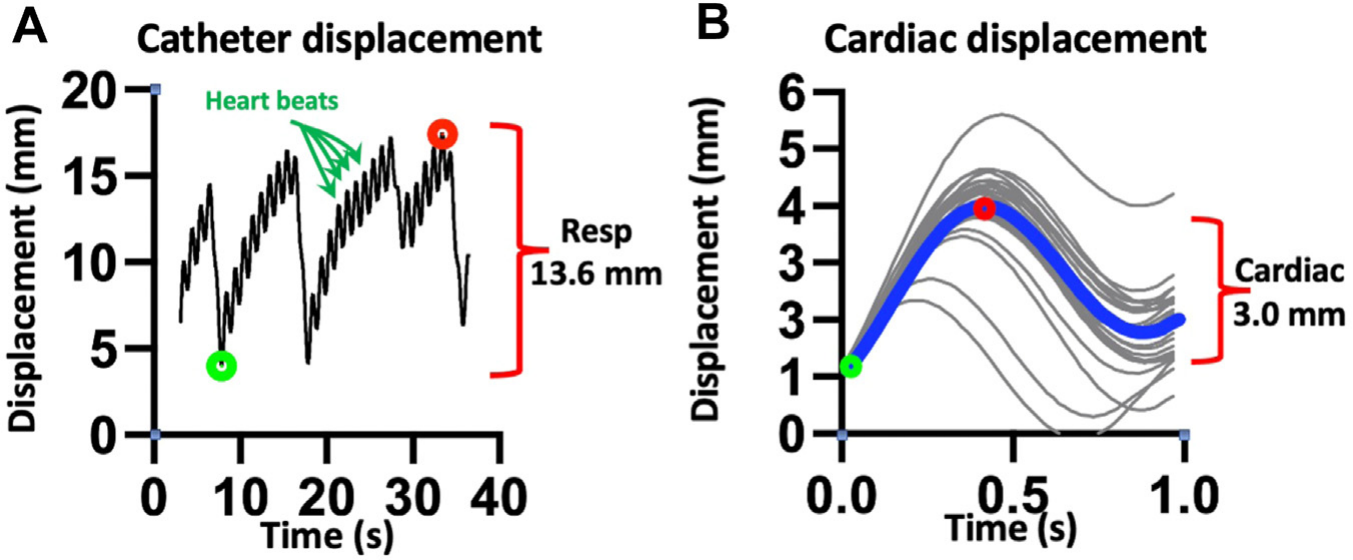
Quantification of cardiorespiratory motion. A: Total catheter displacement as a function of time during an ablation lesion. Two distinct families of oscillations can be appreciated—a high-frequency component corresponding to cardiac contraction and a lower-frequency component corresponding to respiratory motion. In this example, the respiratory component was evaluated at 13.6 mm. B: The total catheter displacement trace is processed to segment the catheter displacements generated by cardiac contraction. In this case, the average cardiac displacement was evaluated at 3.0 mm.

Case files and electroanatomic mapping data from the CARTO software including geometry data were exported. The ablation catheter’s spatial coordinates were used for the analysis. Geometry data were translated into vectorial movement data using an algorithm specifically created using MATLAB (R2023a Update 1; The MathWorks, Inc). During radiofrequency energy delivery, the catheter displacement mirrors the displacement of the endocardial segment in contact with the catheter, which corresponds to the combined cardiac and respiratory displacements or CRM. The catheter spatial displacement over time has 2 distinct components: (1) a lower-frequency component (10–20 cycles/min or 0.15–0.3 Hz) corresponding to the displacement related to respiratory motion and (2) a higher-frequency component (60–100 cycles/min or 1–1.7 Hz) corresponding to the displacement related to cardiac motion (Figure 1). Using an automated algorithm, the catheter trajectory during each ablation lesion was analyzed and the relative contribution of cardiac vs respiratory motion to catheter displacement was calculated. For each ablation lesion, the magnitude of respiratory motion was measured as the maximal excursion of the lower-frequency oscillations (Figure 1A). We used the catheter’s vectorial motion data to calculate the heart rate and segmented each trace to isolate the cardiac contribution to CRM. Each ablation lesion contributed multiple cardiac cycles; unless otherwise stated, the average of all the cycles for each lesion was used for the analysis. We reported 3-dimensional motion data along axes X, Y, and Z (anteroposterior, craniocaudal and mediolateral, respectively), as well as total motion, which reflects maximum 3-dimensional motion amplitude per point taken. All ablation points were performed in sinus rhythm.

## Results

### Study population

The study population consisted of 44 patients who had undergone catheter ablation for VT (Table 1). Twenty-four patients had ICM, whereas 20 patients were ablated for NICM (of which 7 patients were ablated in the right ventricle and 4 patients in the outflow tract regions of the right or left ventricle). Thirty-four (77.3%) patients were male, the average age was 65.8 ± 9.6 years, and body mass index (BMI) was 28.6 ± 5.9 kg/m^2^. The mean left ventricular ejection fraction (LVEF) was 33.5 ± 11.7%. Of the 44 procedures, 20 (45.5%) were performed under general anesthesia and mechanical ventilation (endotracheal intubation), whereas the rest were performed under deep sedation (free breathing).

**Table 1:** Patient and clinical characteristics and total average cardiorespiratory motion data

	Total (N = 44)	ICM (n = 24)	NICM (n = 9)	RV (n = 7)	OT (n = 4)	*P*
Male	34 (77.3)	18 (75)	8 (88.9)	5 (71.4)	3 (75)	.823
Age, y	65.8 ± 9.6	66.0 ± 10.5	70 ± 7.7	63 ± 9.8	61 ± 2.9	.2978
Height, cm	170.9 ± 9.2	170.4 ± 9.0	174 ± 9.4	171 ± 8.9	167 ± 12	.5593
Weight, kg	84.1 ± 20.8	87.0 ± 22.2	86 ± 19	75 ± 16	79 ± 26	.5527
BMI, kg/m^2^	28.6 ± 5.9	29.8 ± 6.1	28 ± 6.3	25 ± 3.8	28 ± 7.2	.4053
LVEF, %	33.5 ± 11.7	28.2 ± 9.3	35 ± 5.2	46 ± 14	40 ± 15	.0016∗
Prior cardiac surgery (yes)	10 (22.7)	9 (37.5)	0 (0)	0 (0)	1 (25)	.53
Breathing (intubated)	20 (45.5)	14 (58.3)	5 (55.6)	1 (14.3)	0 (0.0)	.045∗
Total average cardiac motion, mm	1.62 ± 1.21	1.70 ± 1.26	1.52 ± 1.15	1.64 ± 1.08	1.14 ± 1.02	.0003∗
Total average respiratory motion, mm	12.12 ± 4.10	12.99 ± 4.42	10.68 ± 2.84	10.28 ± 2.83	10.68 ± 3.30	.0126∗

Values are n (%) or mean ± SD. ∗Significant value.

BMI = body mass index; ICM = ischemic cardiomyopathy; LVEF = left ventricular ejection fraction; NICM = nonischemic cardiomyopathy; OT = outflow tract; RV = right ventricle.

### CRM analysis

The average total cardiac and respiratory excursion across the study population was 1.62 ± 1.21 mm and 12.12 ± 4.1 mm, respectively. In all patients, the magnitude of respiratory motion was significantly larger than that of cardiac motion, with the average respiratory/cardiac excursion ratio of 11.45 ± 8.77 (*P* < .001) (Table 2). In other words, respiratory motion was, on average, more than 11 times greater than cardiac motion. However, the cardiac motion component in the outflow tract was significantly less compared with the other subgroups, which led to a an even greater cardiac-to-respiratory-motion ratio in this group compared with the others (ratio > 15). Conversely, the cardiac-to-respiratory-motion ratio was smallest in the right ventricular region (9.39 ± 7.42), due to a relatively larger cardiac component.

**Table 2:** Comparison of respiratory and cardiac motion per cardiomyopathy and ablation area

	ICM (n = 787)	NICM (n = 310)	RV (n = 184)	OT (n = 83)	*P*
Cardiac total excursion, mm	1.70 ± 1.26	1.52 ± 1.15	1.64 ± 1.09	1.14 ± 1.02	.0003∗
Respiratory total excursion, mm	12.99 ± 4.42	10.68 ± 2.84	10.28 ± 2.83	10.28 ± 2.86	<.0001∗
Relation respiratory/cardiac excursion	11.45 ± 8.77	11.61 ± 10.03	9.39 ± 7.42	15.10 ± 10.62	<.0001∗

Values are mean ± SD. *P* values are from analysis of variance testing. ∗Significant value.

ICM = ischemic cardiomyopathy; NICM = nonischemic cardiomyopathy; OT = outflow tract; RV = right ventricle.

When analyzing the respiratory motion component, it was largest in the ICM group (12.99 ± 4.42 mm vs 10.28–10.68 mm in the 3 other groups) (Table 2).

**Figure 2:**
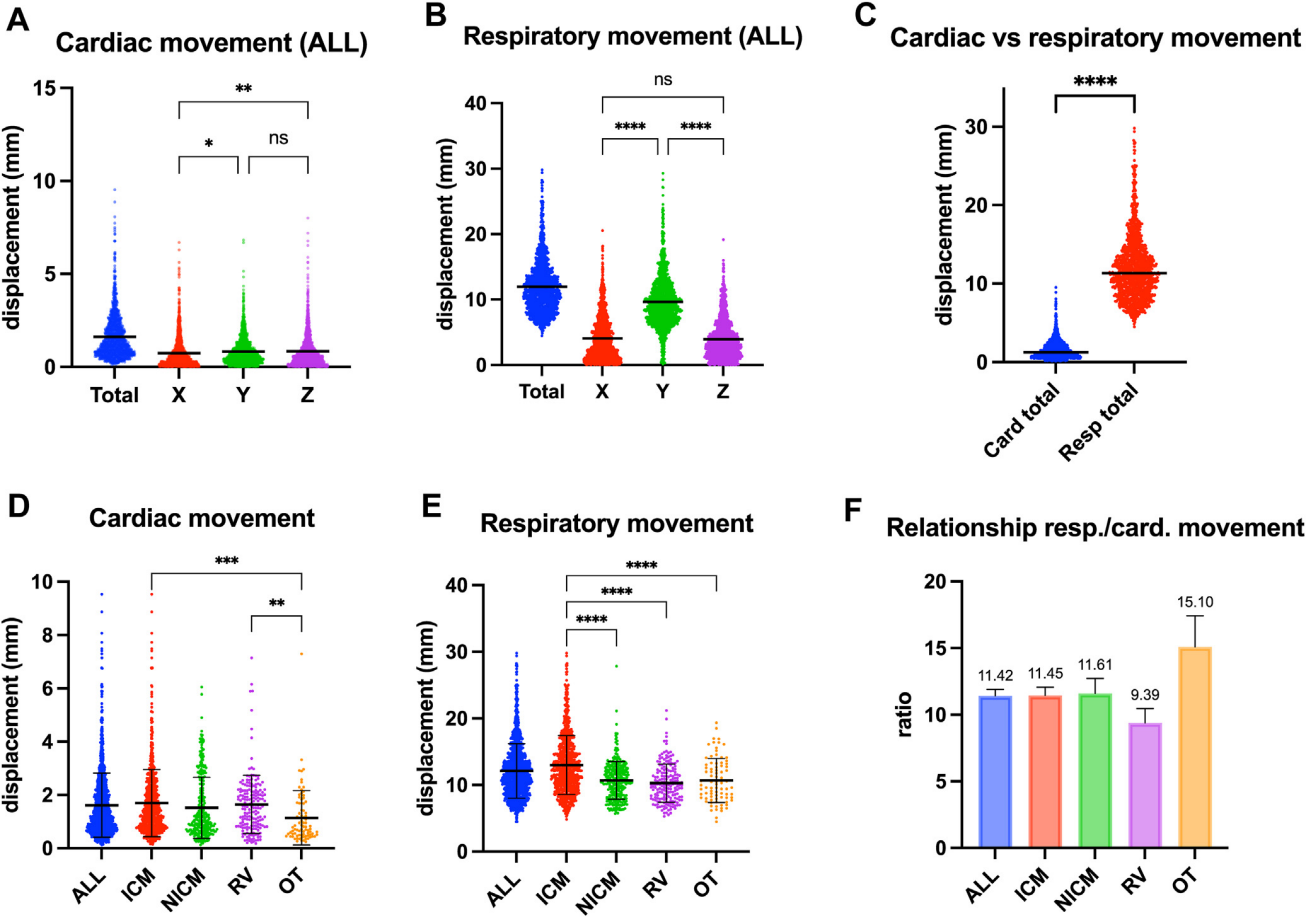
Average cardiorespiratory motion (CRM). Top: Average cardiac (A) and respiratory (B) contribution to CRM per vector direction (X: mediolateral; Y: craniocaudal; Z: anteroposterior) and in total (cardiac vs respiratory) (C). Bottom: Average cardiac (D) and respiratory (E) contribution to CRM per subgroup of cardiomyopathy/ablation area. F: Ratio of respiratory to cardiac contribution to CRM. ∗*P* < .05; ∗∗*P* < .01; ∗∗∗*P* < .001; ∗∗∗∗*P* < .0001. ICM = ischemic cardiomyopathy; NICM = nonischemic cardiomyopathy; ns = not significant; OT = outflow tract; RV = right ventricle.

Overall, cardiac and respiratory motion was most pronounced in the ICM group vs the 3 other subgroups (Table 2, Figure 2). Figure 2 shows the magnitude of the cardiac and respiratory contributions to CRM during 1364 ablation lesions in 44 patients (median 26.5 [interquartile range 20–41.25] lesions per patient). The analysis used to estimate the cardiac vs respiratory components of motion is depicted in a representative example in Figure 1, in which the maximal respiratory displacement was 13.6 mm and the average cardiac displacement was 3.0 mm.

### Direction of CRM

**Figure 3:**
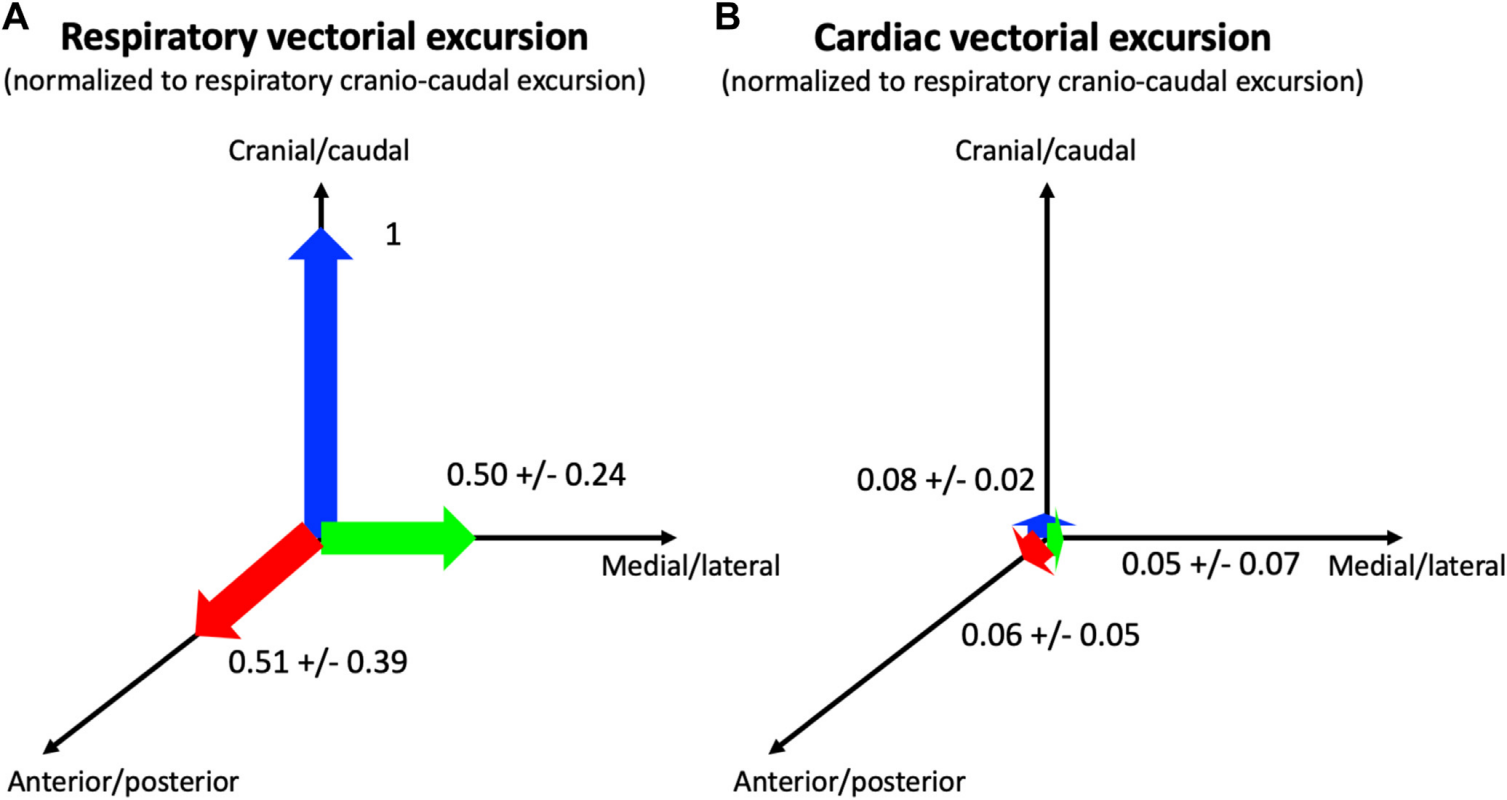
Vectorial cardiorespiratory motion. Average respiratory (A) and cardiac (B) motion along the 3 orthogonal axes. Both are normalized to the largest component (ie, respiratory craniocaudal motion).

Using the catheter displacement’s vectorial information, we assessed the direction of cardiac and respiratory excursion. For each ablation lesion, we decomposed the respiratory and cardiac displacement along the anteroposterior, craniocaudal, and mediolateral dimensions. We found that the craniocaudal component of respiratory motion had the largest relative magnitude (9.66 ± 4.0 mm) and was more than twice as large as the mediolateral (4.09 ± 3.44 mm, *P* < .0001) and anteroposterior displacements (3.96 ± 3.00 mm, *P* < .0001) (Figure 3). Conversely, the magnitude of cardiac excursion was much smaller and, albeit statistically significant, with likely no clinical implication (directional variability in the submillimeter range, varying between 0.75 and 0.84 mm) (Table 3, Figure 3).

**Table 3:** Comparison of respiratory and cardiac motion per direction of movement

	Total	X (mediolateral)	Y (craniocaudal)	Z (anteroposterior)	*P*
Cardiac excursion, mm	1.62 ± 1.21	0.75 ± 0.84	0.83 ± 0.75	0.84 ± 0.91	.0058∗
Respiratory excursion, mm	11.96 ± 4.04	4.09 ± 3.44	9.66 ± 4.00	3.96 ± 3.00	<.0001∗

Values are mean ± SD. *P* values are from analysis of variance testing. ∗Significant value.

ICM = ischemic cardiomyopathy; NICM = nonischemic cardiomyopathy; OT = outflow tract; RV = right ventricle.

### Segmental CRM

**Figure 4:**
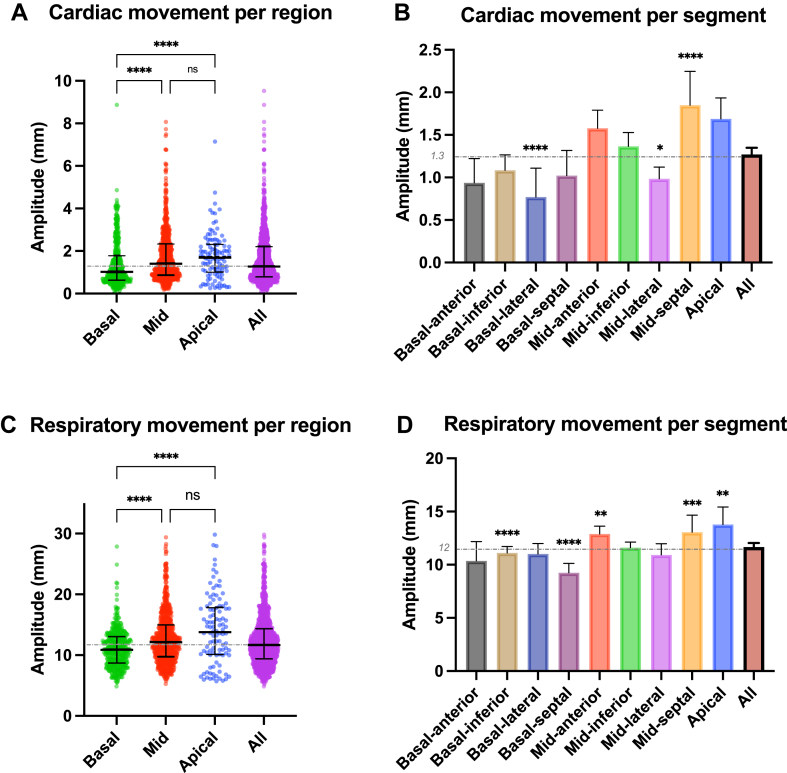
Segmental cardiorespiratory motion. Top: Cardiac displacement per region (basal, midventricular, apical) (A) and per segment (B). Bottom: Respiratory displacement according to region (C), and regional and segmental movement analysis (D) included only left ventricular data. ∗*P* < .05; ∗∗*P* < .01; ∗∗∗*P* < .001; ∗∗∗∗*P* < .0001. ns = not significant.

We segmented the left ventricle into 9 segments (4 basal segments, 4 midventricular segments, and 1 apical segment) to analyze the magnitude of CRM in each segment. We found a significantly larger cardiac movement amplitude from basal to apical segments. However, the absolute difference was small (<0.4 mm) (Figure 4A). On per-segment analysis, basolateral segments had the smallest movement amplitude, whereas midseptal segments had the largest movement amplitude (Figure 4B). Respiratory excursion was sensitive to segmental location in a similar way but with even larger differences in amplitude (Figure 4C). Basal segments had the smallest (11.09 ± 3.11 mm),the midventricular segments intermediate (12.8 ± 4.25 mm), and the apex segments the largest (14.7 ± 5.67 mm) respiratory excursions (*P* < .001). On per-segment analysis, basoseptal had the least respiratory movement, while the apical segment had the largest respiratory movement (Figure 4D).

### CRM vs clinical characteristics

**Figure 5:**
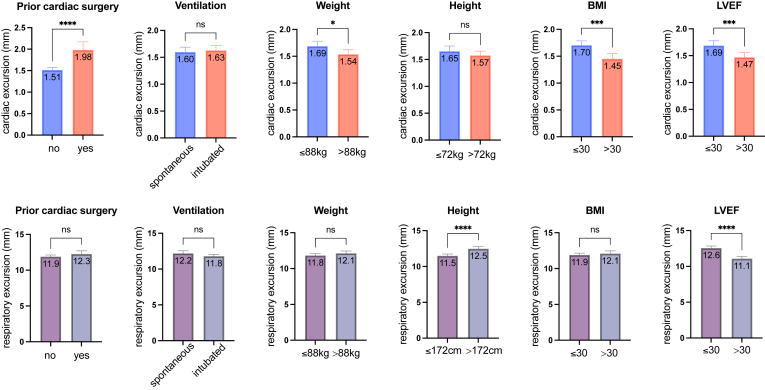
Cardiac and respiratory displacement and their respective association with patient and clinical characteristics. Top: Association of cardiac excursion with patient and clinical characteristics. Bottom: Association of respiratory excursion with patient and clinical characteristics. ∗*P* < .05; ∗∗∗*P* < .001; ∗∗∗∗*P* < .0001. BMI = body mass index; LVEF = left ventricular ejection fraction; ns = not significant.

Cardiac excursions were significantly impacted by prior cardiac surgery status: cardiac excursion was significantly more pronounced in patients who had previous cardiac surgery vs no surgery (1.98 ± 1.63 mm vs 1.51 ± 1.04 mm, *P* < .001). Conversely, higher weight, higher BMI, and higher LVEF were associated with lesser amplitude of cardiac excursion. Notably, differences in motion excursion were very small from an absolute point of view, ranging from 0.15 mm to 0.47 mm (Figure 5, top).

The influence of respiration on CRM, on the other hand, was not associated with prior cardiac surgery. However, a significant association was found between height (taller patients had larger respiratory excursions than smaller patients: 12.47 ± 4.47 mm vs 11.49 ± 3.54 mm, *P* < .001) and LVEF (LVEF >30% led to lower respiratory excursions vs LVEF ≤30%: 11.07 ± 3.86 mm vs 12.56 ± 4.10 mm, *P* < .001) (Figure 5, bottom).

Interestingly, ventilation status (intubated vs spontaneous respiration) had no significant impact on CRM, on neither cardiac nor respiratory excursions.

## Discussion

The main findings of this study are that (1) the size of respiratory displacement is, on average, 11 times larger than cardiac motion; (2) there is regional variability in the magnitude of CRM, with the apical segments moving more than the basal segments; and (3) the amplitude of CRM is modulated by patients’ biometric characteristics. To our knowledge, this is the first systematic evaluation of CRM in patients with clinical characteristics similar to those of patients treated with cardiac radioablation.

### Cardiac radioablation and CRM

The initial experience with cardiac radioablation was highly successful, with 99.9% reduction in VT burden.^2^ However, more recent cohort studies have reported a high rate of clinically significant VT recurrence, often requiring repeat catheter ablation in high-risk patients.^10^ The precise mechanism of action of cardiac irradiation to treat ventricular arrhythmias is not well understood and is a matter of active research. There are important fundamental and technical challenges to optimize cardiac SBRT efficacy and safety.

Delivering the desired dose of radiation to the target while minimizing exposure to surrounding healthy structures is an important objective in any form of radiation therapy. Physiological motion of the target with, for example, breathing imposes additional challenges to treatment planning and delivery. These challenges exist with conventional SBRT of solid tumors (eg, of the lung or adjacent organs).^8^^,^^9^^,^^11^ Strategies have been developed to cope with organ motion during extracardiac SBRT, such as tracking or gating to ensure that the target always stays within the radiation beam and to reduce additional radiation margins.^9^ In SBRT, the planning target volume (PTV) defines the volume of tissue to which the therapeutic dose of radiation will be delivered. In general, the PTV is larger than the actual target to account for uncertainties in treatment planning and delivery, such as organ motion.^12^ Cardiac radioablation imposes additional challenges due to the complex kinetics of the target involving respiratory and also cardiac motions.

### Components of cardiorespiratory heart motion

CRM can be divided into 2 components: a high-frequency, low-amplitude component (cardiac motion) and a low-frequency, high-amplitude component (respiratory motion). Our analysis found that respiratory motion is on average more than 11 times greater than the displacement generated by cardiac contraction. Furthermore, we found that cardiac excursions themselves were very small, at around 1.6 ± 1.2 mm in the target area. Using computed tomography imaging, Ouyang and colleagues^13^ reported an average left ventricular displacement of 0.51–1.2 mm, which is similar to what we found with our method. Using a similar technique but with deep-inspiration breath hold, Wang and colleagues^14^ reported somewhat larger, but qualitatively similar, displacements of 2.3–2.6 mm of the left anterior descending coronary artery as a surrogate for cardiac motion. These studies included patients without significant cardiac disease and did not report segmental CRM; therefore, they may not be applicable to patients undergoing cardiac radioablation. From a clinical standpoint, the magnitude of cardiac motion is close to the limit of resolution of most linear accelerators, such that motion compensation through tracking may only be useful for the respiratory component.^15^ The relatively low intrinsic cardiac motion may indeed best be accounted for by slightly expanding the target volume, as suggested by previous authors.^16^^,^^17^

### Magnitude of respiratory motion

The heart moves with the diaphragmatic contraction during respiration. We observed that displacement of the heart, caused by breathing excursions, was most pronounced in the craniocaudal axis, averaging 9.66 ± 4.00 mm. Our measurements are in line with previously reported values using alternative methods in the range of 10.2 to 16.5 mm.^18^^,^^19^ Meanwhile, we found the anteroposterior and mediolateral respiratory motion to be of significantly lower amplitude, consistent with prior reports.^18^^,^^19^ At present, the target volume is generally expanded symmetrically along all 3 dimensions to account for respiratory displacement of the VT circuit during treatment. Our findings suggest that it would potentially be reasonable to expand the target volume asymmetrically, with larger borders (2:1 ratio) along the craniocaudal direction in such a way to encompass the target within the treatment field while minimizing the exposure to surrounding tissues. Only few studies, such as from Lehmann and colleagues,^20^ reported anisotropic target volume expansion in the animal model. However, the border expansion ratios in this work were not consistent with our findings, which might have been due to the lack of detailed 3-dimensional motion data, such as provided in the present article.

Surprisingly, respiratory cardiac motion was not different between patients under general anesthesia vs sedation. We attribute this to the use of intermittent positive pressure ventilation during general anesthesia, which leads to relevant tidal volume changes over the respiratory cycle. Our results would suggest that these volume changes might not be significantly different from those occurring with spontaneous breathing in sedated patients. This assumption is supported by data on cardiac ablation in patients on jet ventilation vs intermittent positive pressure ventilation: jet ventilation virtually immobilizes the patients’ thorax, eliminating all breathing movements.^21^ This ventilation method was shown in studies to significantly improve catheter stability and ablation outcomes, which was ascribed to reduced movement of the heart.^22^^,^^23^

### Segmental myocardial motion

Interestingly, cardiac and respiratory motion varied significantly across myocardial segments. We found that both, respiratory and cardiac motion, increased from basal to apical, with the apex being subjected to the most important displacement. To our knowledge, this finding has not been previously reported, yet it is important to further refine cardiac radioablation planning, as it suggests that the amount of target volume expansion should be modulated by the target’s location in the heart. Along the same lines, different VT locations may put surrounding structures at more (or less) risk of radiation exposure and potential complications. Detailed motion data allow for a better risk-benefit estimation of expanding treatment volume in different areas of the heart.

### Influence of clinical factors on CRM

We found that patients’ biometric characteristics such as height, weight, and BMI had a significant impact on respiratory motion. Interestingly, the influence was not the same for respiratory and for cardiac motion. Respiratory motion was more pronounced in taller patients, which seems plausible considering the main direction of respiratory movement being craniocaudal. Cardiac motion, in contrast, was negatively influenced by weight and BMI (increased weight and BMI were associated with less movement). A lower LVEF contributed to higher cardiac and respiratory excursions, and the reason for this remains elusive; however, the absolute difference in respiratory heart motion was only very small. Cardiac intrinsic motion was furthermore significantly impacted by a state of prior cardiac surgery, which seemed to increase cardiac motion. However, influence of the mentioned factors on absolute numbers of cardiac and respiratory motion was little, in the submillimeter range for cardiac motion (range 0.15–0.47 mm) and only slightly more for respiratory motion (range 0.99–1.49 mm); therefore, it is likely of little clinical relevance.

### Quantification of CRM to assist in cardiac SBRT planning

In the study by Cuculich and colleagues,^2^ CRM was accounted for by expanding target volume, or internal target volume (ITV), after calculating organ motion by the integration of data from free breathing computed tomography and a respiration-correlated computed tomography (4-dimensional computed tomography). This remains the most common way of quantifying CRM for cardiac radioablation treatment planning. Although clinically practical, this method does not allow for quantification of the cardiac contribution to CRM, neither segmental nor regional differences. A different approach was used in the study of Neuwirth and colleagues^3^: the ICD lead of the patient was tracked to compensate respiration, while cardiac contraction was taken into account through ITV calculation. A recent study from Lydiard and colleagues^16^ examined the possibility of tracking both respiratory and cardiac motion by a dynamic multileaf collimator during a radioablative procedure for atrial fibrillation.^16^ This technique showed a high accuracy; however, it requires a complex setup using an MRI-Linac with real-time magnetic resonance imaging target tracking of atrial myocardium.

To further improve future cardiac radioablation procedures and assist with ITV and PTV calculations, displacement of the heart needs to be quantified precisely, but the analysis of cardiorespiratory heart motion is challenging. Different approaches have been described. Recently, the motion of atrial myocardium was investigated using real-time MRI tracking of the tissue.^16^^,^^18^^,^^19^ However ventricular myocardial motion might differ, as the ventricles are much thicker and have different contractile characteristics compared with the atria. Bellec and colleagues^24^ observed that cardiorespiratory ITV could be estimated by using 2 extreme cardiac phases combined with 10 respiratory phases from 4-dimensional computed tomography, rather than using a single cardiac phase. Ouyang and colleagues^13^ studied the motion of cardiac substructures including the left and right ventricles by contouring them and following the position of the respective centroid. Wang and colleagues^14^ looked at the displacement of the left anterior descending artery as a surrogate during deep-inspiration breath-hold to assess cardiac contraction motion. One could assume, though, that cardiac motion analyzed through these methods is representative neither for the motion of all the different ventricular wall segments nor for a myocardial region affected by scarring. Arrhythmogenic areas, which are the target of cardiac SBRT in VT treatment, are usually located in regions of scar. Improving the understanding of cardiac motion in these specific areas is paramount to better guide treatment volume calculations or evaluate the necessity of tracking methods for cardiac SBRT.

The approach used in the present study to evaluate cardiac movement differs from previous approaches by the fact that we measured motion directly in the target area and at the endo/myocardial surface by using geometrical data of an ablation catheter. The 3-dimensional mapping system tracks the catheter position with much higher temporal and spatial resolution than usual computed tomography scans can deliver. Furthermore, the analysis runs efficiently using already existent patient data from previous electrophysiological studies without exposing the patient to additional radiation-based diagnostic studies. It allows for patient-specific CRM quantification and could therefore easily be implemented to assist in cardiac SBRT planning.

One previous study from Roujol and colleagues^25^ used a slightly different approach to quantify CRM. As with our method, Roujol and colleagues quantified CRM by analyzing data from an electroanatomic mapping system and separated the cardiac and respiratory component by looking at the different frequency bands. For their analysis, Roujol and colleagues used points from point per point mapping data. Hence, in contrast to our study, they did not have contact force to ensure contact with the heart and indeed rejected many data points because of supposed instability. Furthermore, each mapping point recorded data for only 2.5 seconds, which sometimes may not cover a whole respiratory cycle, and may also lead to less reliable data compared with a longer recording time (typically 30–45 seconds for ablation lesions). Last, mapping data were collected from the whole ventricle, whereas we only collected data from the target area. Roujol and colleagues observed less cardiac motion in scar area; this underscores the necessity to define CRM in these areas in more detail, what we attempted with our method. However, for NICM there might be less variability between scar regions and the rest of the heart because scar in NICM is often not transmural and with more diffuse distribution.

### Limitations

First, the total number of patients was relatively low. However, the total number of ablation points available for analysis was large and represented scar locations across the left ventricle. Second, we analyzed only a surrogate to heart motion (ie, catheter tip movement) and not the heart motion itself. Even though we can assume that catheter tip movement comes very close to true CRM, there is always the potential for inherent measurement imprecisions of the employed system. We cannot rule out that the catheter contact to the endocardial surface could have led to some variation in myocardial excursion based on the amount of force applied. However, based on our experience with intracardiac echocardiography, myocardial movement is not visibly impacted under usual ablation circumstances. Also, the catheter tip only represents the movement of the endomyocardial or epicardial part of the heart and may not account for differences between endo- and epicardial movement in a defined area, although we would expect them to be small and not clinically significant. A strength of our method was that the collected data points were situated in the target areas for catheter ablation, which would also represent the main target for radioablation. Although motion data from these regions are the most relevant for cardiac SBRT, we cannot generalize this to myocardial motion in other (healthy) regions, as our method only enabled us to collect data in areas of ablation. It is also worth mentioning that we did not use any immobilization devices during our ablation procedures. Hence, our results regarding respiratory movements might not apply to patients treated in centers that treat using an abdominal belt. However, there is no consensus on treatment modalities in cardiac SBRT, and many teams treat without using these compression devices.^7^ Furthermore, we had only limited data on right ventricular and outflow tract locations, and the performed analyses on these subgroups are therefore of limited value but may indicate the need to further quantify CRM in these specific locations. Finally, even though the catheters all used contact force, and ablation is usually done with relatively stable and reliable contact with the cardiac surface, we cannot exclude that the operator accepted lower or intermittent contact for some ablation points.

## Conclusion

CRM is primarily determined by respiratory displacement and is modulated by the location of the target and the patient’s biometric characteristics. A better understanding of the magnitude and directionality of CRM can help in optimizing PTV delineation for cardiac radioablation. Patient-specific quantification of CRM may allow to decrease the treatment volume and expose less healthy tissue to radiation while delivering the therapeutic dose to the target.
